# Comparison of Performance between White Light Imaging and Narrow Band Imaging in Distinguishing Neoplastic and Non-neoplastic Colorectal Polyps

**DOI:** 10.34172/mejdd.2025.405

**Published:** 2025-01-31

**Authors:** Kaka Renaldi, Lily Verawati, Hasan Maulahela, Nur Rahadiani, Aulia Rizka, Ari Fahrial Syam, Dyah Purnamasari, Chyntia Olivia Maurine Jasirwan, Dicky Levenus Tahapary

**Affiliations:** ^1^Division of Gastroenterology, Pancreatobiliary, and Gastroenterological Endoscopy, Department of Internal Medicine, Faculty of Medicine-University of Indonesia, Dr. Cipto Mangunkusumo National Hospital, Jakarta; ^2^Department of Internal Medicine, Faculty of Medicine-University of Indonesia, Dr. Cipto Mangunkusumo National Hospital, Jakarta; ^3^Department of Pathological Anatomy, Faculty of Medicine-University of Indonesia, Dr. Cipto Mangunkusumo National Hospital, Jakarta

**Keywords:** White light imaging, Narrow band imaging, Performance, Colorectal polyps

## Abstract

**Background:**

White light imaging (WLI) is the current standard colonoscopy technique for diagnosing colorectal polyps in Indonesia. Various endoscopic imaging techniques have been developed to improve the accuracy of diagnosing colorectal polyps, one of which is narrow band imaging (NBI). We conducted a diagnostic study comparing the performance of NBI against WLI in distinguishing neoplastic from non-neoplastic colorectal polyps.

**Methods:**

This was a diagnostic study that analyzes endoscopic pictures of colorectal polyps in patients who underwent colonoscopy using the WLI and NBI techniques. Previously collected biopsy tissue specimens were re-examined by a single pathologist.

**Results:**

There were 117 subjects analyzed, and the proportion of subjects with neoplastic polyps was 65.8%. Common indications for colonoscopy were hematochezia (24.8%) and abdominal pain (23.9%). WLI showed moderate inter-observer reliability (kappa value=0.591), while NBI showed significant reliability (kappa value=0.674). NBI demonstrated better sensitivity (84.4%; 95% CI 74.4%–91.7%) and accuracy (78.6%; 95% CI 70.1%–85.7%) compared with WLI (sensitivity 74%; 95% CI 62.8%–83.4% and accuracy 71.8%; 95% CI 62.7%–79.7%). However, the specificity was the same (67.5%; 95% CI 50.9%–81.4%).

**Conclusion:**

NBI has better performance than WLI in distinguishing neoplastic and non-neoplastic colorectal polyps.

## Introduction

 According to World Health Organization (WHO) GLOBOCAN 2020, colorectal cancer’s global incidence ranks as the third most common cancer and is the second leading cause of cancer-related deaths after lung cancer.^1^ In Indonesia, colorectal cancer also ranks among the top five most common cancers with a high mortality rate.^2^ When diagnosed at an early stage, colorectal cancer has a very high 5-year survival rate. Nearly 95% of colorectal cancers develop from colorectal polyps that grow slowly over several years and do not exhibit significant symptoms. Therefore, by detecting and managing colorectal polyps earlier, it is possible to prevent colorectal cancer.^3,4^

 Colorectal polyps are classified histologically as neoplastic or non-neoplastic.^5,6^ Colonoscopy can detect precancerous lesions early on.^3^ The current standard for colonoscopy, including in Indonesia, is the white light imaging (WLI) technique. Several new endoscopic techniques have been developed to enhance and improve the accuracy of detecting colorectal polyps, and one of these techniques is narrow-band imaging (NBI).^7,8^

 Since its introduction in 2005,^9^ numerous studies have tested the performance of NBI in detecting and determining the characteristics of colorectal polyps.^8,10-13^ However, existing studies have shown diverse performance outcomes. One of the reasons for this diversity is the inappropriate use of the term “serrated polyp,” leading to inconsistencies in classifying serrated polyps.^13,14^

 To improve diagnostic accuracy and reduce inter-operator variability in diagnosing colon lesions using NBI, the NBI International Colorectal Endoscopic (NICE) classification system was proposed in 2012.^7,10,15^ However, the NICE classification system does not include sessile serrated polyps.^7,15^ As a result, the Workgroup Serrated Polyps and Polyposis (WASP) classification system was developed to distinguish adenomas, hyperplastic polyps, and sessile serrated polyps endoscopically.^16^ This study aims to evaluate the performance of WLI as the current standard endoscopic technique in Indonesia and NBI using the NICE and WASP classification systems in distinguishing neoplastic and non-neoplastic polyps in the colorectal.

## Materials and Methods

###  Study Design

 Thiswas a single-center diagnostic study to evaluate the performance of WLI and NBI in differentiating neoplastic and non-neoplastic colorectal polyps.

###  Study Population

 This study utilized retrospective data from 117 patients who underwent colonoscopy at the gastrointestinal endoscopy center of Dr. Cipto Mangunkusumo National Hospital from January to December 2022. Inclusion criteria included adult patients aged ≥ 18 with colorectal polyps detected using WLI and NBI techniques and subsequently biopsied for tissue examination. Exclusion criteria encompassed inadequate quality of endoscopic pictures and incomplete medical record data.

###  Sampling Methods

 Endoscopic pictures of colorectal polyps obtained using the WLI and NBI techniques were gathered. Endoscopy experts assessed these photos for their quality. Two separate endoscopy experts used Images of good quality to determine the type of polyp as either neoplastic or non-neoplastic using NICE and WASP classification systems. Neoplastic polyps included adenoma, serrated adenoma, and colorectal carcinoma, while non-neoplastic polyps included hyperplastic polyps, inflammatory polyps, and hamartomatous polyps. An expert pathologist re-examined the tissue biopsy specimens that had previously been collected.

###  Outcome Measures

 The primary outcome measure was the sensitivity, specificity, positive predictive value, negative predictive value, likelihood ratio, accuracy, and area under the curve of the receiver operating characteristic (AUC ROC) of WLI and NBI in distinguishing neoplastic from non-neoplastic colorectal polyps.

###  Statistical Analyses

 The research data were analyzed using the Statistical Package for the Social Sciences (SPSS) version 24. The data was presented as text, tables, and images as needed. Descriptive categorical data analysis was presented as proportions (percentages). Numeric data with a normal distribution was presented as the mean with standard deviation, while numeric data with a non-normal distribution was presented as the median with range.

 To ensure the reliability of the results obtained from WLI and NBI, interobserver reliability analysis was performed using kappa statistics. The sensitivity, specificity, positive predictive value, negative predictive value, positive likelihood ratio, negative likelihood ratio, accuracy, and the area under the curve of the receiver operating characteristic (AUC ROC) of WLI and NBI were compared to evaluate their diagnostic performance in differentiating between neoplastic and non-neoplastic colorectal polyps.

## Results

###  Characteristics of the Patients

 Among 1298 patients who underwent colonoscopy for various indications, 171 patients were found to have colorectal polyps and met the inclusion criteria. After applying the exclusion criteria, 117 research samples were analyzed in this study. WLI determined that 59.8% (n = 70) of the subjects had neoplastic polyps. The percentage increased to 66.7% (n = 77) when using NBI. The histopathology results showed that neoplastic polyps were found in 65.8% (n = 77) of subjects, as shown in Table 1.

**Table 1:** Patient characteristics

**Characteristic**	**n=117**
Age (y), median (min-max)	61 (23–85)
Sex, n (%)	
Male	60 (51.3)
Female	57 (48.7)
WLI, n (%)	
Neoplastic polyps	70 (59.8)
Non-neoplastic polyps	47 (40.2)
NBI, n (%)	
Neoplastic polyps	78 (66.7)
Non-neoplastic polyps	39 (33.3)
Histopathology, n (%)	
Neoplastic	77 (65.8)
Adenoma	48 (41)
Tubular adenoma	43 (36.8)
Villous adenoma	0 (0)
Tubulovillous adenoma	5 (4.3)
Serrated adenoma	4 (3.4)
Sessile serrated adenoma	3 (2.6)
Traditional serrated adenoma	1 (0.9)
Carcinoma	25 (21.4)
Adenocarcinoma	25 (21.4)
Squamous cell carcinoma	0 (0)
Non-neoplastic	40 (34.2)
Hyperplastic polyps	19 (16.2)
Inflammatory polyps	19 (16.2)
Hamartomatous polyps	2 (1.7)
Number of polyps, n (%)	
1	66 (56.4)
≥ 2	51 (43.6)
Size of polyps, n (%)	
< 10 mm	75 (64.1)
≥ 10 mm	42 (35.9)
Site of polyps, n (%)	
Caecum	2 (1.7)
Ascending colon	10 (8.5)
Transverse colon	10 (8.5)
Descending colon	13 (11.1)
Sigmoid colon	18 (15.4)
Rectum	24 (20.5)
Multiple sites	40 (34.2)
Colonoscopy indications, n (%)	
Hematochezia	29 (24.8)
Abdominal pain	28 (23.9)
Constipation	20 (17.1)
Chronic diarrhea	12 (10.3)
Melena	5 (4.3)
Unintentional weight loss	3 (2.6)
Anemia	1 (0.9)
Others	19 (16.2)
Family history of GI malignancy, n (%)	
Yes	4 (3.4)
No	82 (70.1)
Missing data	31 (26.5)

GI = gastrointestinal.

###  Interobserver Reliability Analysis

 The interobserver reliability analysis results for WLI yielded a kappa value of 0.591, indicating moderate concordance, while for NBI, a kappa value of 0.674 was obtained, indicating significant concordance.^17^ Both observers are endoscopy experts with similar years of experience and expertise in using WLI and NBI techniques. Therefore, this study can use the results from either one of the observers. This study utilizes the results from the first observer.

###  Comparison of Performance between WLI and NBI 

Table 2 shows a comparison of the diagnostic performance between WLI and NBI. It demonstrates that NBI has higher sensitivity than WLI with the same specificity. NBI also exhibits better positive predictive value, negative predictive value, positive likelihood ratio, negative likelihood ratio, and accuracy than WLI.

**Table 2:** Comparison of diagnostic performance between WLI and NBI

**Variable**	**WLI (95% CI)**	**NBI (95% CI)**
Sensitivity	74% (62.8%–83.4%)	84.4% (74.4%–91.7%)
Specificity	67.5% (50.9%–81.4%)	67.5% (50.9%–81.4%)
Positive predictive value	81.4% (73.4%–87.5%)	83.3% (76%–88.8%)
Negative predictive value	57.5% (46.7%–67.6%)	69.2% (56.2%–79.8%)
Positive likelihood ratio	2.3 (1.4–3.6)	2.6 (1.6–4.1)
Negative likelihood ratio	0.4 (0.3–0.6)	0.2 (0.1–0.4)
Accuracy	71.8% (62.7%–79.7%)	78.6% (70.1%–85.7%)

**Figure 1:**
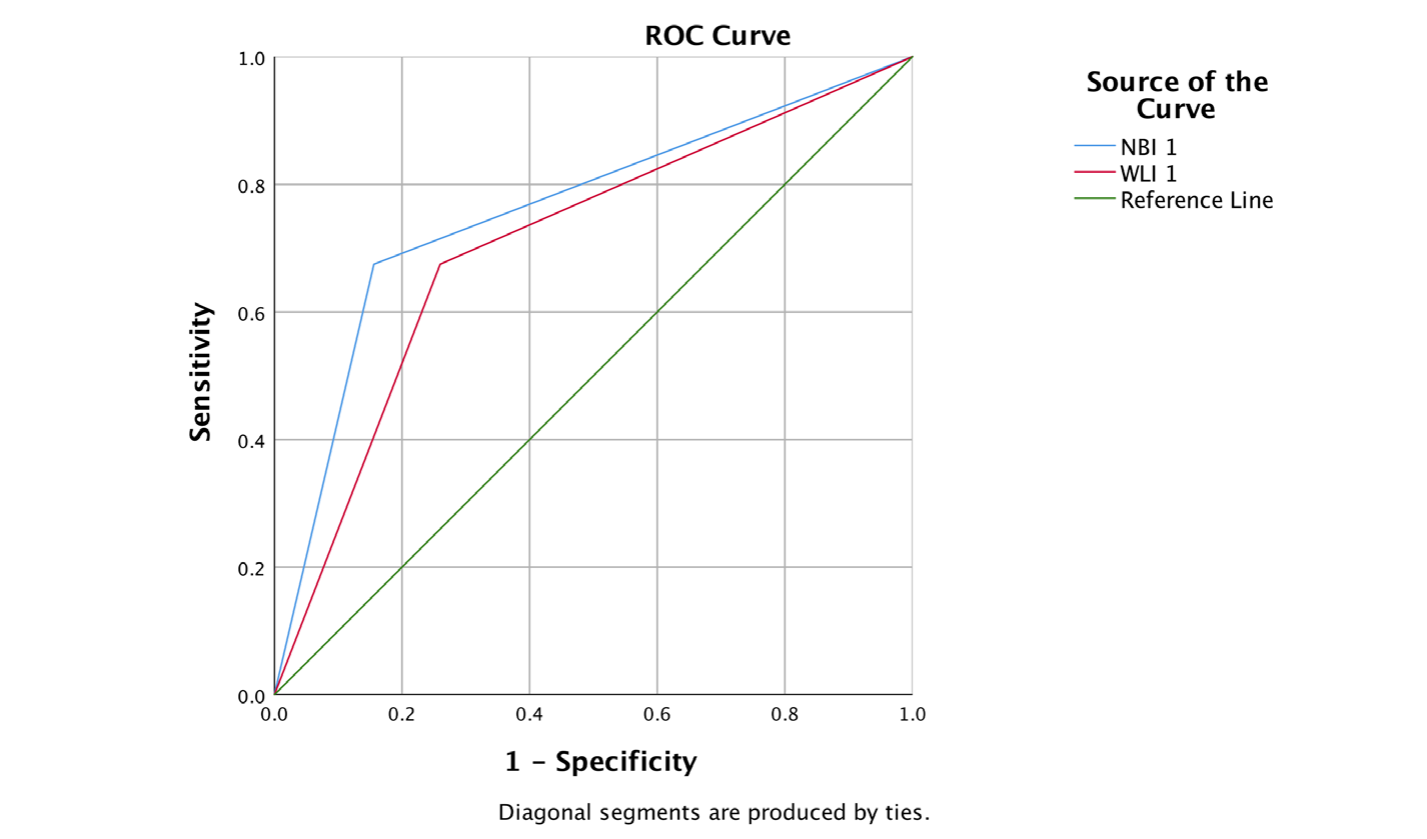
Comparison of ROC curves for WLI and NBI

Figure 1 shows a comparison of ROC curves between WLI and NBI. The area under the curve for the NBI examination is larger (AUC = 0.760; 95% CI 0.672–0.834) compared with WLI (AUC = 0.708; 95% CI 0.616–0.788).

## Discussion

 Colorectal adenoma and cancer cases were more common in men (23.1% and 13.7%, respectively) than in women (21.4% and 7.7%, respectively) in this study. The average age of the subjects was 61 years old. A meta-analysis by Wong et al of 70 studies conducted in Asia, America, and Europe also reported similar findings. The prevalence of colorectal neoplasia was higher in men, as well as in the age group of ≥ 50 years old.^18^

**Figure 2:**
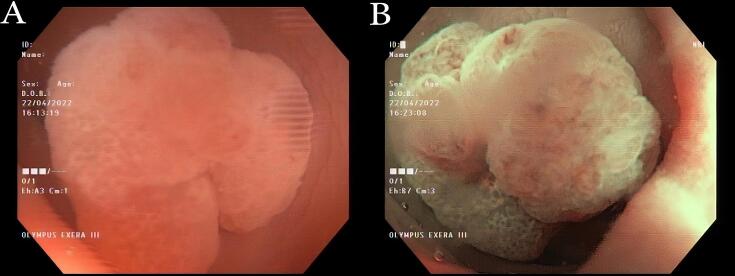
Sessile serrated adenoma on WLI (A) and NBI (B)

 Most colorectal polyps obtained were adenomas (41%). In China, it was found that 55.8% of colorectal polyps were also adenomas.^19^ Similarly, in the United States, adenomatous polyps are the most common pathological findings (n = 8,305; 59.9%) in patients aged ≥ 50 years,^20^ as found in this study. By combining the NICE and WASP classification systems,^16^ 75% of serrated adenoma cases in the study could be accurately identified, as shown in Figure 2.

**Figure 3:**
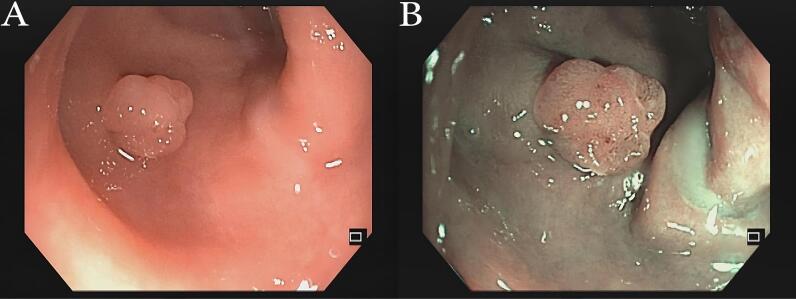
Neoplastic colorectal polyp showing histopathology of an adenocarcinoma on WLI (A) and NBI (B)

 There were 21.4% cases of colorectal adenocarcinoma among all colorectal polyp samples. This figure is significantly higher compared with the prevalence of colorectal cancer in the general population, which is 0.4%.^18^ This might be due to the invasion of cancer into the submucosa of the colon, which cannot be easily detected macroscopically,^21^ leading it to be assessed as colorectal adenoma polyp, as in one of the samples in this study (Figure 3).

 Most colorectal polyps found in this study were single polyps (56.4%) and smaller than 10 mm (64.1%). There is variation in the data regarding the number and size of colorectal polyps in various studies in Asia,^22^ America,^20^ and Africa.^23^ This variation can be attributed to differences in the population at risk in each country, changes in lesion characteristics during the course of the disease, and the presence or absence of clinical symptoms as an indication for colonoscopy.^20^

 In this study, most adenomas were found on the left side of the colon (67.9%). This differs from other studies that report significantly more adenomas on the right side of the colon (69.4%).^24^ This difference may be attributed to the fact that polyps on the right side of the colon are more challenging to detect due to the anatomical shape of the right colon.^25^

 In Indonesia, national health insurance does not cover colorectal cancer screening tests. As a result, 99% of the patients in this study underwent colonoscopy based on clinical indications. The most common indications were lower gastrointestinal bleeding (24.8%) and abdominal pain (23.9%). These findings are consistent with other studies in Asia,^26,27^ except in countries with medical insurance programs for colorectal cancer screening, where most patients are asymptomatic (46%).^28^

 The interobserver reliability of NBI is better than that of WLI in this study. This result was found in a study with a prospective design as well.^29^ This study indicates that NBI has better sensitivity and accuracy than WLI in distinguishing neoplastic and non-neoplastic colorectal polyps but has the same specificity. However, the difference in the AUC between NBI and WLI (0.052) is not statistically significant (*P* = 0.204).

 Both WLI and NBI are optical diagnostics that are subjective and highly influenced by the quality of the captured images. However, this study utilized retrospective data in the form of standard-resolution documentation photos of colonoscopies that were not originally intended for research purposes. This means that suboptimal image capture of polyps could affect interpretation. Additionally, determining the histology of colon polyps through endoscopy can sometimes be challenging due to various lesion characteristics and the shape of the colon where the lesion grows. Even for the same lesion, there can be variations in terms of brightness, size, shape, and texture.^30^ These factors explain why the performance of both techniques in this study was lower than in previous studies.

 This study is the first in Indonesia to examine the diagnostic value of NBI as an advanced endoscopic technique in distinguishing neoplastic colorectal polyps from non-neoplastic ones. To avoid errors in classifying sessile serrated polyps when interpreting pictures of colorectal polyps taken using NBI, this research used the NICE and WASP classification systems. These techniques make it easy to assess the macroscopic characteristics of polyps. By combining both classification systems, 75% of serrated adenoma cases in the study could be accurately identified.

 This study has several limitations. Like other retrospective studies, it shares similar shortcomings, including incomplete patient characteristic data due to missing information in medical records. The documentation photos of polyps in this study are from retrospective data and were not originally taken for research purposes, leading to potential issues with image quality, such as standard resolution and inconsistent image positioning, which could impact interpretation. In this study, most of the polyp tissue samples were obtained through biopsy rather than polypectomy, so the histopathological results may not accurately represent the true prevalence of colorectal polyps. Additionally, this study was conducted in a tertiary referral hospital by experienced endoscopy experts, so its findings may not be easily generalized to community settings or less-experienced endoscopy practitioners.

## Conclusion

 In conclusion, NBI performs better than WLI in distinguishing neoplastic and non-neoplastic colorectal polyps.
